# Effect of lentivirus-mediated miR-182 targeting FGF9 on hallux valgus

**DOI:** 10.7150/ijms.50984

**Published:** 2021-01-01

**Authors:** Wei-Lin Zhang, Duo-Yi Zhao, Wei Zhao, Yan Cui, Qin Li, Zhi-Yu Zhang

**Affiliations:** 1 Department of Orthopedics, the Fourth Affiliated Hospital of China Medical University, No.4 East Chongshan Road, Shenyang, 110032, P.R. China; 2 Center for Translational Medicine, the Fourth Affiliated Hospital of China Medical University, No.4 East Chongshan Road, Shenyang, 110032, P.R. China

**Keywords:** hallux valgus, FGF9, miR-182, osteoblast, alkaline phosphatase

## Abstract

The pathogenesis of hallux valgus is not clearly understood. However, genetics research about hallux valgus is rare. Therefore, the present study aimed to explore the pathogeny of hallux valgus from the perspective of genetics. Human samples were collected from normal bone tissue and hallux valgus region bone tissue. The bone samples were studied using real time-PCR, western blot and immunohistochemical. Lentivirus-mediated miR-182 transfected osteoblasts and tested the expression of FGF9 mRNA with real time-PCR. To test alkaline phosphatase activity, number of calcium nodules and proliferation of osteoblast with enzymatic activity analysis, calcium nodules stained and MTT assay. We found that (1) FGF9 expressed in hallux valgus region bone tissue was significantly higher than normal bone tissue. (2) miR-182 expression levels in hallux valgus region bone tissue were notably lower than those of normal bone tissue. (3) miR-182 could negatively regulate the expression of FGF9 in osteoblasts. (4) FGF9 may enhance osteoblasts proliferation. We have demonstrated that miR-182 promotes the formation of bone by targeting FGF9, implicating an essential role of miR-182 in the etiology of hallux valgus. Moreover, miR-182 might potentially be a therapeutic target for hallux valgus treatment.

## Introduction

Hallux valgus deformity refers to lateral displacement of lateral deviation in the first metatarsophalangeal joint, which is commonly referred to as a bunion, is a foot disease with a higher incidence in women. It is a complex anatomical deformity that is challenging to treat. Hallux valgus makes the patient very painful and it may lead to foot, ankle, or knee joint deformity 1. Although the research on hallux valgus has been paid more attention in recent years, it mainly focuses on anatomical structure and clinical treatment, and the pathogenesis of thumb hallux valgus is rare. The main pathological changes that occur in a hallux valgus are osteophyte formation and articular capsule thickening. However, the molecular biology mechanism of hallux valgus is not clearly understood.

Previous studies have shown that fibroblast growth factors (FGFs) may stimulate the proliferation of the bone tissue of the first plantar toe joints providing a new direction in the investigation of hallux valgus disease pathology 2. FGFs contains the two large intron sequences and three exons area, which is one of the characteristics of FGF family factor. The size of the protein-coding regions of FGFs genes ranges from 5 to 100 kb 3. FGFs play an important role in the process of endochondral ossification and subperiosteal osteogenesis 4. FGF9 is an important member of FGFs. FGF9 is widely distributed in various tissues of the human body. It is involved in a variety of physiological and pathological processes such as bone development, blood vessel formation, embryonic development, damage repair, cell apoptosis, and tumor growth 5-7. Its existence can effectively promote mitosis and cell growth. FGF9 could form dimers in solution with a K(d) of 680 nm 8 and activate fibroblast growth factor receptor 2 (FGFR2) to promote osteogenesis 9,10. Investigating the regulation of FGF9 in hallux valgus may be useful for understanding the development and pathogenesis of this disease.

Recent studies have contributed to our understanding of the pathogenesis of hallux valgus through genetic predisposition 11,12. However, gene-related studies of this disease are rare. In our study, we focus on the miRNAs expressed in hallux valgus. Hundreds of miRNAs that regulate protein-encoding genes have been identified. These miRNAs play the role of regulators in cell proliferation, differentiation, apoptosis, as well as organ development and inflammatory diseases 13,14. Recent studies have demonstrated that miRNA-182 (miR-182) can regulate FGF9 in nerve cells 15,16.

In this study, we explored the relationship between the expression of FGF9 in the bone tissue of the hallux valgus region and the formation of valgus. Moreover, we studied the effect of miR-182 on the expression of FGF9 in the bone tissue of the hallux valgus region.

## Methods

### Preparation of the lentivirus vector

The lentiviral vector (Genechem, shanghai, China) labeled with green fluorescent protein (GFP) encoding hsa-pre-miR-182 or reverse complement sequence of hsa-miR-182 was employed for hsa-miR-182 up-regulation and hsa-miR-182 knockdown. Blank lentiviral vector labelled with GFP was employed as negative control.

### Specimen collection

Ten hallux valgus that were removed during surgical procedures were used in this study (36 women and 4 men; age range, 20-75 years). Patient consent for removal of the tissue was obtained before surgery. Samples were taken for morphological observation and extraction of RNA. 20 cadaver specimens as control (12 women and 8 men; age range, 20-60 years). Cadaver specimens from the knee in patients with severe trauma blood supply to the leg amputation in the car accident.

### Immunohistochemistry

Tissue sections (5μm) were incubated with rabbit anti-hsa FGF9 (1:500; Abcam) primary antibody. Horseradish peroxidase-labeled goat anti-rabbit (1:400; Santa Cruz Biotechnology) secondary antibody was used. Semi-quantitative analysis was performed at 200× magnification per visual field (0.145 mm^2^) for FGF9 extravasation, using imaging software (ImagePro Plus 6.0; Media Cybernetics, Bethesda, MD, USA). The mean IOD values were analyzed and averaged. The semi quantitative analysis of immunohistochemical results on the basis of the positive cell percentage ratio and tinting strength.-and ± judged as the negative, + and + + judged as positive, the positive cells : 0% recorded as 0 points, ≤25% recorded as 1 points, 26-50% recorded as 2 points, 51-75% recorded as 3 points, >75% recorded as 4 points. Coloring intensity: no color recorded as 0 points, Light yellow recorded as 1 point, Claybank recorded as 2 points, Brown recorded as 3 points. Two results are combined: 0 points for (-), 2-3 points for (±), 4-5 points for (+),6-7 points for (+ +).

### Cell culture

The hFOB cells, kindly provided by Dr. M. Subramaniam 17, were cultured in a 1:1 mixture of Hamo's F12 Medium Dulbeccoo's Modified Eagle's Medium without phenol red (Sigma, St. Louis, MO, USA), supplemented with 10% fetal bovine serum (FBS) (HyClone; Thermo Fisher Scientific, Fremont, CA, USA), in a humidified 5% CO2 atmosphere at 37°C with the medium being changed every other day.

### Gene expression analysis using real time-Polymerase Chain Reaction (real time-PCR)

Total RNA was extracted from the samples using Trizol reagent (Invitrogen, USA) according to manufacturer's instructions and quantified spectrophotometrically at 260 nm with acceptable 260/280 ratios between 1.8 and 2.0. The RNA quality was also checked by 1% agarose gel electrophoresis, stained with 1 μg/mL ethidium bromide. Residual genomic DNA was removed by incubating RNA with RNase- free DNase (Promega, USA). Total RNA was reverse transcribed using a reverse-transcription kit (TaKaRa, China) according to the manufacturer's protocols. Real-time PCR was performed on ABI Prism 7900HT Fast System (Applied Biosystems, USA) using SYBR Premix Ex TaqTM II (TaKaRa, China). Amplifications were carried out in a total volume of 20 μL and cycled 40 times after initial denaturation (95°C for 30 s) with the following parameters: 95°C for 5 s and 60°C for 30 s. Primers sequences were listed in Table 1, and β-action was used as an internal control. The relative mRNA expression was quantified through a comparison of the cycle threshold (Ct) values. The experimental data were processed using the 2-ΔΔCt method: ΔΔCt = (Ct target-Ct internal control) experiment group-(Ct target-Ct internal control) normal control group. Each experimental group was repeated 3 times.

### Western blotting

After treatment, the cells were extracted with lysis buffer (150 mm NaCl, 1% NP-40, 0.1% SDS, 2 μg/mL aprotinin, 1 mm PMSF) for 30 min at 4°C. The supernatants were centrifuged at 12,000 g for 15 min at 4°C. The supernatant containing total protein was harvested. Aliquots containing 50 μg of proteins were separated by a 12% SDS-PAGE and transferred to PVDF membranes at 60 V or 40 V for 2 h at low temperature. The membranes were soaked in blocking buffer (5% skimmed milk) for 2 h. Subsequently, proteins were detected using primary antibodies at 1:500 or 1:1000 dilution for overnight at 4°C, then visualized using anti-goat or anti-rabbit IgG conjugated with peroxidase (HRP) at 1:6000 or 1:8000 dilution for 2 h at room temperature. The EC3 Imaging System (UVP Inc. Upland, CA, USA) was used to catch up the specific bands, and the optical density of each band was measured using an Image J software (NIH, Bethesda, MD, USA). The rate between interesting proteins and GAPDH of the same sample was calculated as relative content and expressed graphically.

### Immunofluorescence

After treatment, the cells were fixed with 4% paraformaldehyde at room temperature for 15 min. After washing with PBS, cells were permeabilized with 0.2% Triton X-100 for 5 min. Sections were incubated in a blocking buffer containing 5% BSA for 30 min at room temperature, followed by incubation with anti-FGF9 (1:200) antibody overnight at 4 °C. Secondary antibodies labeled with fluorescein (1:500, Abcam) were applied for 120 min. After incubating with 0.1% DAPI for 10 min and another washing step with PBS, images were captured on a wide field fluorescent microscope (Olympus, Japan). Each experiment was repeated three times (n=90 cells) and the fluorescence intensities of the images were quantified with ImageJ software.

### MTT assay

For cell proliferation studies, osteoblast was seeded at a celldensity of 105 cells/well on the samples placed in the wells and also on the polystyrene surface of the well in a 24-well tissue culture plate (TCP). The plate material was taken as the positive control in this study. The proliferation of osteoblast on the sample surfaces was measured after 24 h, 48 h, 72 h by MTT assay. The samples with attached cells were incubated in a mixture of 360 μL of PBS (phosphate buffer saline) and 40 μL MTT solution (5 mg/mL in PBS) for 4 h at 37 °C in 5% CO2 atmosphere. The intense red colored formazan derivatives formed were dissolved in 400 μL dimethyl sulfoxide for 15 min and the absorbance was measured with a microplate reader at a wave length of 590 nm.

### ALP Detection

The cells from each group were thrice-washed with PBS and lysed with 0.1% Triton X-100 using 3 cycles of freezing and thawing to verify that the cells were completely lysed. The cell lysates were centrifuged at 15 000 g for 5 min at 4℃, and the supernatant was collected. 50 μl of cell lysate was transferred to a 96-well plate and 150 ml of pNPP (Sigma-Aldrich, Inc.Irvine, UK) was added to each well as substrate for 20 min at 37℃. p-NP was quantified based on the spectrophotometric absorbance at 405 nm. ALP activity was normalized to the total protein concentration for each sample using a BCA protein assay (Pierce Biotechnology).

### pFL-*FGF9* 3'-UTR vector construction and luciferase reporter assay

The 3'-UTRs segments of human *FGF9* mRNA containing the putative miR-182 binding sequence were inserted into pGL3-control (Promega, Madison, WI, USA), respectively. HEK 293 cells were cotransfected with 0.8 μg firefly luciferase reporter vector containing the target site, 100 nM miR-182 double stranded mimics or miR-control (Ambion, Austin, TX, USA) and 0.04 μg Renilla luciferase control vector (pRL-TK-Promega), using Lipofectamine 2000 (Invitrogen). Assays were performed 48 h after transfection, using the dual luciferase reporter assay system (Promega, Madison, WI, USA). Firefly luciferase activity was normalized to Renilla luciferase activity. The mutations on miR-182 binding sites in human FGF9 3'-UTRs were generated using the Quick Change XL Site-Directed Mutagenesis kit (Stratagene, La Jolla, CA, USA). Each mutation consisted of replacing four consecutive base pairs at the 3' region of the site.

### Osteogenic induction

1 ml 0.1% Gelatin Solution was added into each well of 6 well plates and shook gently to cover the bottom. They were placed at room temperature for 30 min and then Gelatin Solution was discarded and let the plates air-dry. The cells were cultured in the coated 6 well plates at 37°C with 5% CO2 for 24 h. The medium was discarded gently and 2 ml OriCell TM Sprague-Dawley (SD) Rat Mesenchymal Stem Cell Osteogenic Differentiation Medium was added into them. The inducing medium was changed for every 3 days and they were induced for 21 days. After that the cells were fixed and stained with Alizarin red. The cells were washed with PBS and fixed with 4% formaldehyde for 30 min. Then they were washed with PBS for 2 times and stained with Alizarin red for 3-5 min at room temperature. They were observed with inverted microscope.

### Statistical analysis

Two-group comparisons were performed using Student's t-test. Multiple group parameters comparisons were performed using one-way analysis of variance followed by Turkey's post-test. A P value less than 0.05 was considered statistically significant. The statistical analysis was performed using the SPSS statistical package (SPSS, Chicago, IL, USA).

## Results

### Correlation between FGF9 and miR-182 in hallux valgus

**Figure 1:**
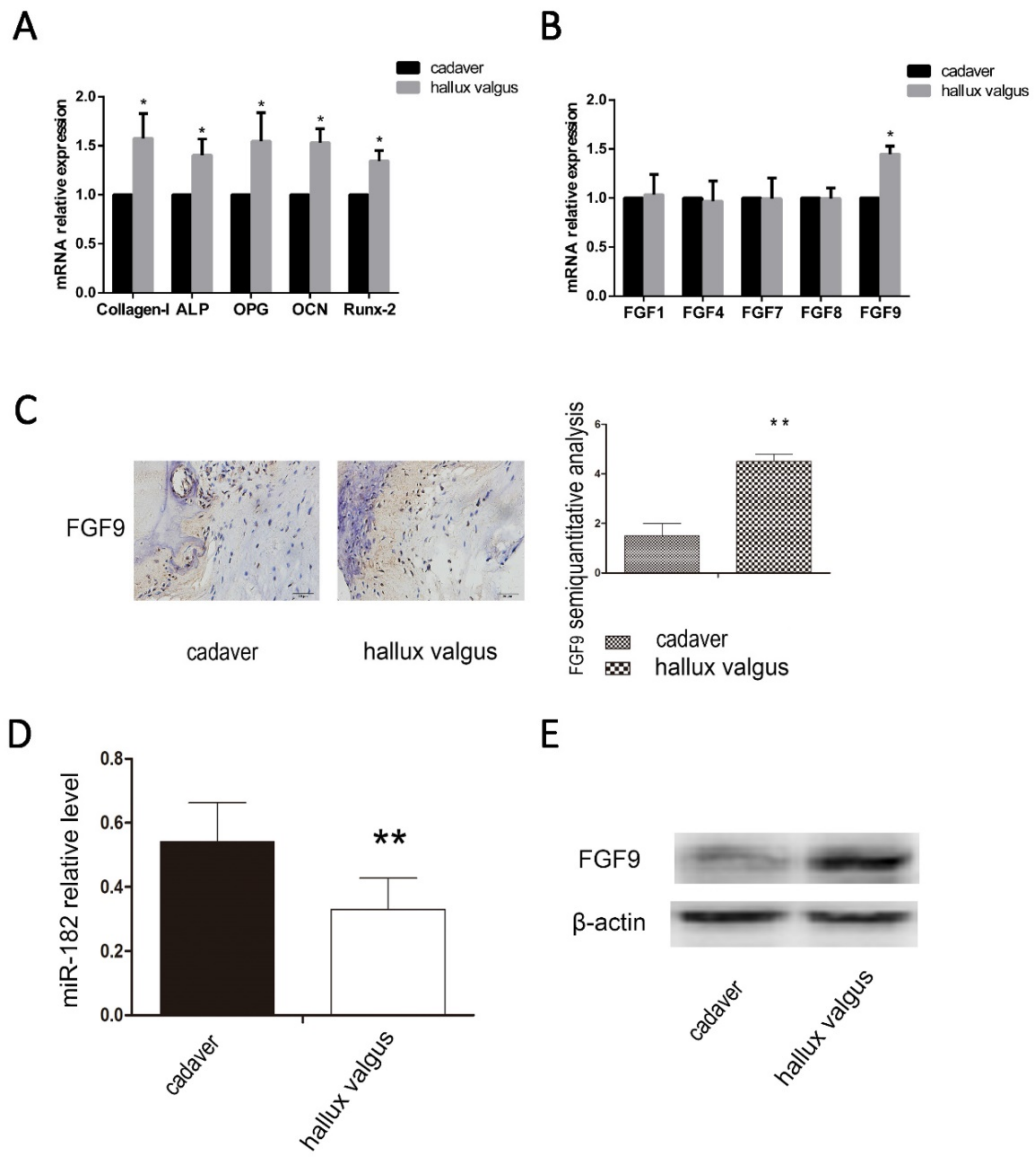
Correlation between FGF9 and miR-182 in hallux valgus. (A)RT-PCR analysis of Collagen-I, ALP, OPG, OCN, and Runx-2. (B) RT-PCR analysis of FGF1, FGF4, FGF7, FGF8, FGF9. (C) The situation of FGF9 expression in the first metatarsal using immunohistochemical method. Hallux valgus group was significantly stronger than cadaver group. Scale bars, 20μm. FGF9 semi-quantitative immunohistochemical analysis showed that Hallux valgus group was significantly stronger than cadaver group. (B) real time-PCR for the expression of miR-182 in Hallux valgus patients and cadaver. (C) Western blot analysis shows expression of FGF9 in Hallux valgus group is stronger than cadaver group. n=40 Hallux valgus group, n=20 cadaver group. Data are means ± SD. *P < 0.05, **P < 0.01.

We analyzed the osteophyte in the first plantar toe joint of 40 hallux valgus patients and the first plantar bones of 20 cadavers in the same position using immunohistochemistry, western blotting and real-time PCR. We examined the mRNA levels of osteogenic capability biomarkers Collagen-I, ALP, OPG, OCN and Runx-2, we found osteogenic hyperfunction in the first plantar toe joint of hallux valgus patients (Figure 1A). Then we examined the mRNA levels of FGF family, FGF1, FGF4, FGF7, FGF8, FGF9 and found the high mRNA level of FGF9 in hallux valgus patients (Figure 1B). We found that the expression of miR-182 was elevated in normal tissues relative to that of hallux valgus patients. Contrastingly, FGF9 levels were elevated in the hallux valgus patients relative to normal tissues (Figure 1C-E).

### miR-182 inhibits FGF9 expression by directly targeting his 3'-UTRs

**Figure 2:**
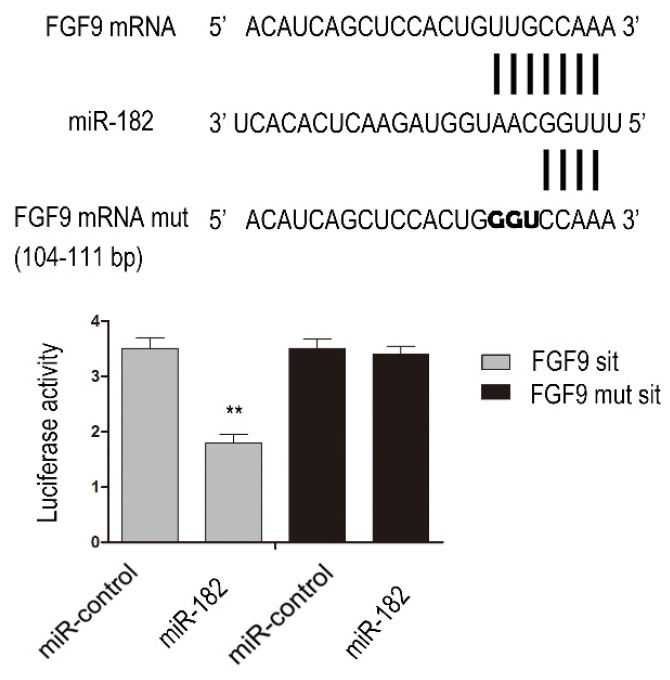
miR-182 can inhibit FGF9 via directly targeting his 3'-UTRs. Complementarity between miR-182 and the putative hFGF9 3'-UTR target site. hFGF9 mut indicate the FGF9 mRNA 3'-UTRs with three consecutive mutation sites in miR-182 binding sites. The relative luciferase activities show hFGF9 3'-UTR 104-111 bp site is binding site of miR-182. n=3 per group. Data are means ± SD. *P < 0.05, **P < 0.01.

To determine whether FGF9 is direct targets of miR-182, we performed luciferase reporter assay. Given that there was a potential binding site in the 3'-UTR of hFGF9 with miR-182, we validated the binding sites. Consequently, the relative luciferase activity of FGF9 104-111 bp site was distinctly diminished (P<0.01) in cells co-transfected with miR-182 double-stranded mimics. In addition, mutation with three consecutive base pairs of the miR-182 binding sites of FGF9 site manifestly abolished the suppression of luciferase activity due to miR-182 over-expression. This shows fully convincingly that miR-182 inhibits FGF9 expression by directly targeting his 3'-UTRs (Figure 2).

### miR-182 regulates FGF9 in osteoblasts

**Figure 3:**
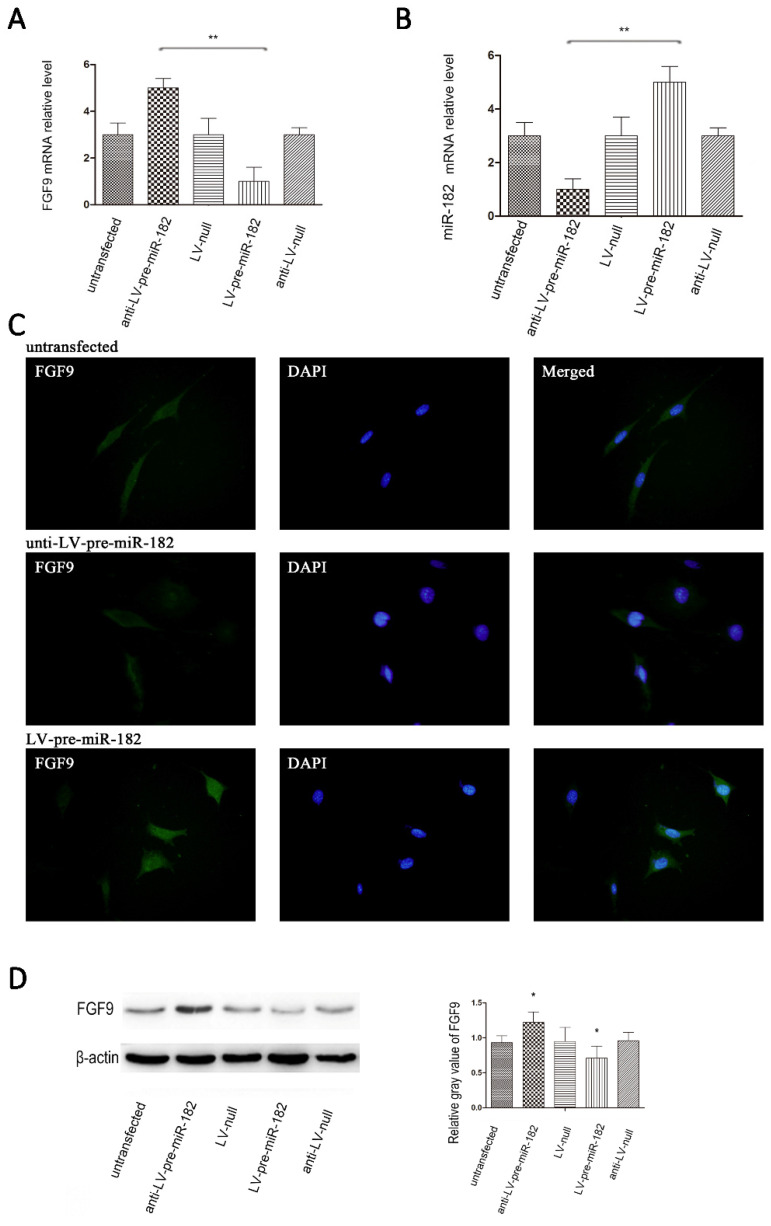
miR-182 regulates FGF9 in osteoblasts. (A) Lentivirus expressing miR-182 could be stably transfected into osteoblasts. real time-PCR analysis showed that miR-182 was down-regulated while FGF9 mRNA is down-regulated; in addition miR-182 is up-regulated while FGF9 mRNA was up-regulated. (B) miR-182 regulates FGF9 in osteoblasts. Western blot analysis shows expression of FGF9 protein in ant-LV-pre-miR-182 group is stronger than LV-pre-miR-182 group. No statistically significant difference between the other groups. (C) Immunofluorescence of FGF9 in hFOB1.19 cells. (D)Western blot analysis of FGF9 expression in transfected hFOB1.19 cells. n=5 per group. Data are means ± SD. *P < 0.05, **P < 0.01.

After establishing the miR-182 inhibits FGF9 expression by directly targeting his 3 '-UTRs, we will verify whether the miR-182 can regulate FGF9 in osteoblast. Transfection with the GFP expressing lentiviral vector at a multiplicity of infection (MOI) of 10 resulted in high-level GFP expression in osteoblasts. Up-regulation of miR-182 resulted in repression of FGF9, whereas knockdown of miR-182 led to over-expression of FGF9 in osteoblasts. In contrast, transfecting osteoblasts with a blank lentiviral control vector had nosignificant effects on the expression of FGF9 in these cells. In addition, we examined FGF9 levels by immunofluorescence, we found that FGF9 protein expression enhanced with knockdown of miR-182. When up-regulate the expression of miR-182, the expression of FGF9 protein reduced (Figure 3).

### The influence of FGF9 on osteogenic potential

**Figure 4:**
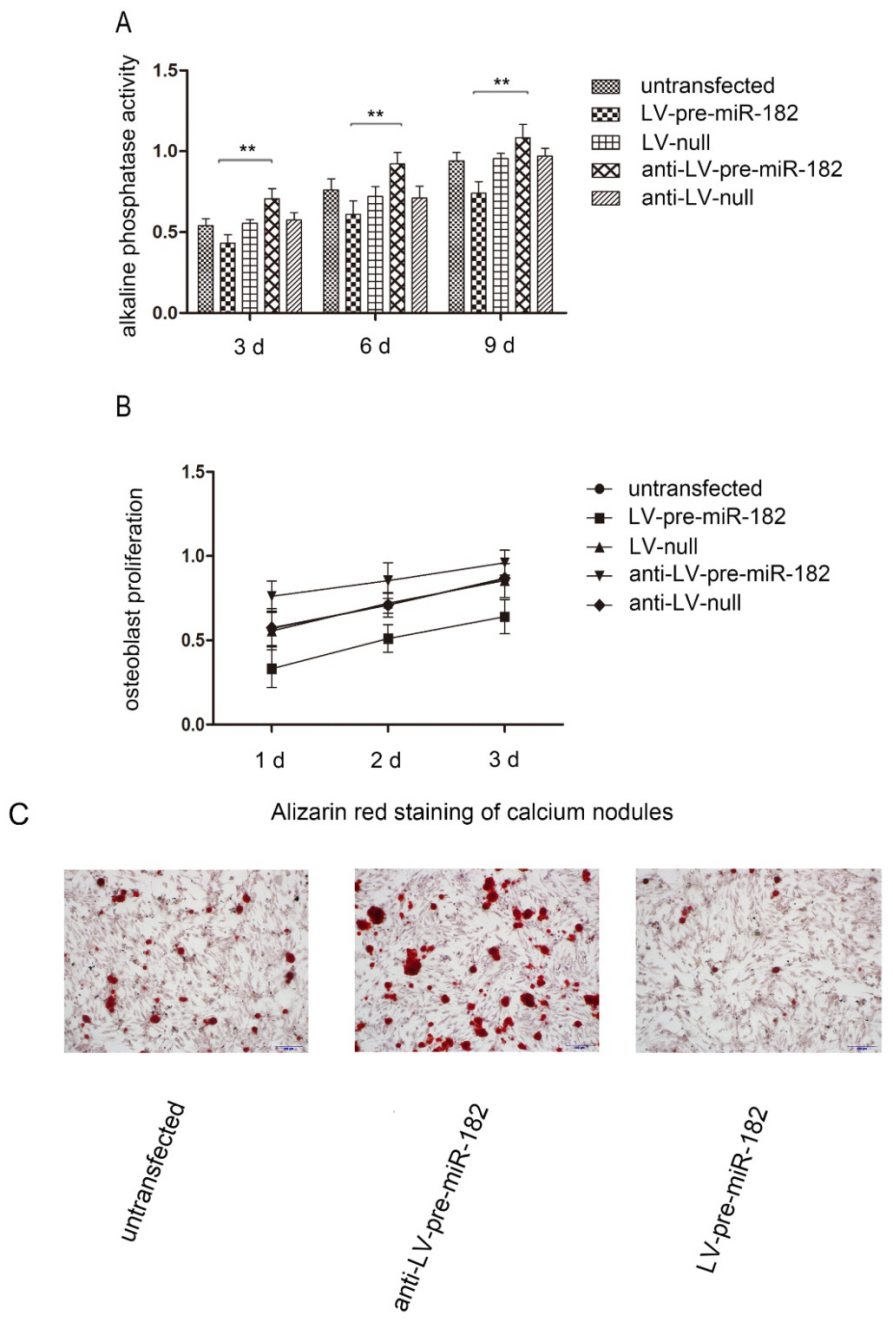
The influence of FGF9 on alkaline phosphatase and osteoblast proliferation. (A) Alkaline phosphatase activity analysis shows that the activity in LV-pre-miR-182 group is significantly lower than the anti- LV-pre-miR-182 group. n=5 per group. Data are means ± SD. *P < 0.05, **P < 0.01. (B) The MTT assay showed that osteoblast proliferation in LV-pre-miR-182 group is significantly lower than the anti- LV-pre-miR-182 group. And (C), in staining calcium nodules, the phenomenon has been verified. n=5 per group. Data are means ± SD. *P < 0.05, **P < 0.01.

In order to observe FGF9 for osteogenesis effects in osteoblast, we carried on the alkaline phosphatase activity, number of calcium nodules and osteoblast proliferation test in osteoblast. With changing FGF9 levels, alkali phosphatase expression, number of calcium nodules and osteoblast proliferation rate was also changed. Evaluation of osteoblast proliferation using an MTT assay showed that as FGF9 expression increased, so did the rate of cell proliferation. Similarly, increasing FGF9 levels increased the detection of alkaline phosphatase activity. In staining calcium nodules, the phenomenon has been verified. Conversely, when FGF9 expression decreased, alkaline phosphatase activity, number of calcium nodules and osteoblast proliferation were diminished (Figure 4).

## Discussion

Hallux valgus is a very common disease. But the molecular biology mechanism of hallux valgus is not clearly understood. In this study, we explored the function of miR-182 and FGF9 in hallux valgus region bone tissue, and verified these findings in osteoblasts.

Excessive osteophyte formation is the main factor causing hallux valgus and inflammation, but the underlying causes need to be further elucidated. By analyzing the tissue of hallux valgus patients and amputated patients, we found that the osteogenic indexes of hallux valgus patients were significantly increased, which confirmed our conjecture. Previous studies have pointed out that FGF stimulation of bone tissues that overlie the head of the first metatarsal is a potential inducement of the hallux valgus and bunion formation ^2^. Therefore, we hypothesized that FGF may be the key to solving this problem. We found a significant increase in FGF9 expression in the FGF family by tissue examination in patients with hallux valgus. FGF9 is involved in bone formation and development. Existing research shows that FGF9 plays an important role in multiple synostosis syndromes (SYNS) 18 and promotes the formation of bone 19. FGF9 can combine with FGFR-2 and up-regulate the process of subperiosteal ossification. We verified the conjecture by measuring the expression of FGF9 at hallux valgus region. The result shows that FGF9 expressed in hallux valgus region bone tissue was significantly higher than normal bone tissue.

Then we focus on the miRNAs expressed in hallux valgus. It has been previously reported that miR-182 plays a role in a wide variety of diseases, including liver cancer, lung tumor and breast cancer 20,21. There is evidence to show that miR-182 can inhibit the activity of osteoblasts 22. However, no previous study has investigated the relationship between miR-182 and hallux valgus. For the first time, we show of the biological connection between miR-182 and hallux valgus. miR-182 expression levels in hallux valgus region bone tissue were notably lower than those of normal bone tissue. That providing a new direction for future therapies.

We consider the main pathogenic factor of hallux valgus to be osteophyte formation. Excess osteophyte formation and enhancement of osteogenesis function as well as increased bone density has a strong correlation 23. We chose indicators such as alkaline phosphatase, number of calcium nodules and osteoblast proliferation to observe how FGF9 affects osteogenesis in osteoblasts. The results showed that FGF9 enhanced the osteoblasts proliferation. To our knowledge, this is the first time that it has been demonstrated that FGF9 has a direct function in osteoblasts.

Some issues need to be further explored in future research. Because there are not many studies on hallux valgus, there is no relatively recognized model in vivo and in vitro to study the pathological mechanism of hallux valgus. Therefore, our study involves many problems such as no in vitro experiment and no purposefulness. However, our study has sufficient sample size and starts from the core pathological mechanism of hallux valgus, so we have reason to believe that This study can provide a new research direction for the pathological mechanism of hallux valgus.

## Conclusion

We have demonstrated for the first time that FGF9 is a novel target of miR-182 and that miR-182 promotes the formation of bone by targeting FGF9, implicating an essential role of miR-182 in the etiology of hallux valgus. Moreover, miR-182 might potentially be a therapeutic target for hallux valgus treatment.

## Figures and Tables

**Table 1:** Primer sequences used in real- time PCR experiments

Gene	Primer sequence 5'-3'
Collagen-I	F: AGAGCTTCGGCAGCAGGA C: CTTATAGCAGTTCTGCCTGC
ALP	F: AACATCAGGGACATTGACGTG R: GTATCTCGGTTTGAAGCTCTTCC
OPG	F: GCGCTCGTGTTTCTGGACA R: AGTATAGACACTCGTCACTGGTG
OCN	F: CACTCCTCGCCCTATTGGC R: CCCTCCTGCTTGGACACAAAG
Runx-2	F: CCT TCCAGACCAGCAGCAG R: TCCGTCAGCGTCAACACCA
FGF1	F: CTCTTTAGTCTTGAAAGCGCC R: TAAACTTCTCGGTCAGGGC
FGF4	F: GGAGTGGTGAGCATCTTTG R: GAAGAAAGCCGAGCCATAG
FGF7	F: TGGGCACTATATCTCTAGCTTGC R: GGGTGCGACAGAACAGTCT
FGF8	F: AGACGGACACCTTTGGAAGC R: TGCCTTTGCCGTTGCTCTTGG
FGF9	F: GCATCCATGGTGTGCCAGTGAAACAGCAGA R: GTAACCATGGCAGAGGACTCGGCTTTTGGA
β-actin	F: GACAGGATGCAGAAGGAGATTACT C: TGATCCACATCTGCTG GAAGGT
